# Formulation Strategies to Improve the Bioavailability of Poorly Absorbed Drugs with Special Emphasis on Self-Emulsifying Systems

**DOI:** 10.1155/2013/848043

**Published:** 2013-12-26

**Authors:** Shweta Gupta, Rajesh Kesarla, Abdelwahab Omri

**Affiliations:** ^1^Department of Pharmaceutics, Parul Institute of Pharmacy, Limda, Waghodia, Vadodara, Gujarat 391760, India; ^2^Department of Chemistry & Biochemistry, Laurentian University, 935 Ramsey Lake Road, Sudbury, ON, Canada P3E 2C6

## Abstract

Poorly water-soluble drug candidates are becoming more prevalent. It has been estimated that approximately 60–70% of the drug molecules are insufficiently soluble in aqueous media and/or have very low permeability to allow for their adequate and reproducible absorption from the gastrointestinal tract (GIT) following oral administration. Formulation scientists have to adopt various strategies to enhance their absorption. Lipidic formulations are found to be a promising approach to combat the challenges. In this review article, potential advantages and drawbacks of various conventional techniques and the newer approaches specifically the self-emulsifying systems are discussed. Various components of the self-emulsifying systems and their selection criteria are critically reviewed. The attempts of various scientists to transform the liquid self-emulsifying drug delivery systems (SEDDS) to solid-SEDDS by adsorption, spray drying, lyophilization, melt granulation, extrusion, and so forth to formulate various dosage forms like self emulsifying capsules, tablets, controlled release pellets, beads, microspheres, nanoparticles, suppositories, implants, and so forth have also been included. Formulation of SEDDS is a potential strategy to deliver new drug molecules with enhanced bioavailability mostly exhibiting poor aqueous solubility. The self-emulsifying system offers various advantages over other drug delivery systems having potential to solve various problems associated with drugs of all the classes of biopharmaceutical classification system (BCS).

## 1. Introduction

Various strategies have been widely investigated to enhance the bioavailability of poorly absorbed drugs in order to increase their clinical efficacy when administered orally. It is estimated that between 40% and 70% of all new chemical entities identified in drug discovery programs are insufficiently soluble in aqueous media [1, 2]. The increase in the proportion of poorly soluble candidates is frequently attributed to improvements in synthesis technology, which has enabled the design of very complicated compounds, and a change in discovery strategy from a so-called phenotypic approach to a target-based approach [3]. Various physicochemical properties which contribute to the poor solubility of various drugs include their complex structure, size, high molecular weight, high lipophilicity, compound H-bonding to solvent, intramolecular H-bonding, intermolecular H-bonding (crystal packing), crystallinity, polymorphic forms, ionic charge status, pH, and salt form [4].

Lipinski's rule of five has been widely proposed as a qualitative predictive model for the assessment of absorption of poorly absorbed compounds. In the discovery setting “the rule of 5” predicts that poor absorption or permeation is more likely when there are more than 5 H-bond donors, 10 H-bond acceptors, the molecular weight is greater than 500, and the calculated Log P is greater than 5. The rule of five only holds for compounds that are not substrates for active transporters and efflux mechanisms [5]. Thus, *in vivo* assessment of new drug candidates in animal model is performed to assess the absorption of drug. Poorly absorbed drugs pose a challenge to the formulation scientists to develop suitable dosage form which can enhance their bioavailability.

Broadly, poorly soluble drugs can be formulated in three different forms to overcome the challenge of poor absorption—crystalline solid formulations, amorphous formulations, and lipid formulations [6].

### 1.1. Crystalline Solid Formulations

Modification of the physicochemical properties such as salt formation and micronization of the crystalline compound to increase the surface area and thus dissolution may be one approach to improve the dissolution rate of the drug. Particle size of about 2–5 *μ*m can be achieved by micronization using air-jet mill. The nanocrystal technology can reduce the crystalline particle size to 100–250 nm using ball-milling [7], dense gas technologies [8], and so forth. However, these methods have their own limitations. For instance, salt formation of neutral compounds is not feasible. Particle size reduction may not be desirable in situations where poor wettability and handling difficulties are experienced for very fine powders [9].

### 1.2. Amorphous Formulations

Amorphous formulations include “solid solutions” which can be formed using a variety of technologies including spray drying and melt extrusion [9–11]. Amorphous formulations may include surfactants and polymers providing surface activity during dispersion.

Other formulation strategies which are most popularly adopted to enhance the bioavailability of such drugs include the complexation with cyclodextrins [12], formulation of polymeric conjugates [13], nanoparticles, solid lipid nanoparticles (SLN) [14], use of permeation enhancers, and surfactants [15].

### 1.3. Lipid Formulations

In recent years, a great deal of interest has been focussed on lipid based carrier systems. The most popular approach is the incorporation of the active poorly water soluble component into inert lipid vehicles such as oils, surfactant dispersions [16], solid dispersions, solid lipid nanoparticles, emulsions, microemulsions, nanoemulsions, self-emulsifying formulations (SEF), micro/nanoemulsifying formulations [17], and liposomes [18].  Table 1 provides a brief indication of the main formulation strategies and the main advantages and disadvantages of each approach [6].

## 2. Lipid Formulations

Lipid formulations for oral administration of drugs are a diverse group of formulations having a wide range of properties. The utility of solubilizing lipid-based formulations for improving the gastrointestinal (GI) absorption of poorly water-soluble, hydrophobic drugs is well documented in the literature. These generally consist of a drug dissolved in a blend of excipients (5 classes of excipients) with wide variety of physicochemical properties ranging from pure triglyceride oils, mono- and diglycerides, and substantial proportion of lipophilic or hydrophilic surfactants and cosolvents.  Table 2 gives the broad classification system for various lipid formulations [19].

The primary mechanism of action by which a lipid formulation leads to improved bioavailability is usually avoidance of the slow dissolution process which limits the bioavailability of hydrophobic drugs from solid dosage forms. Preferably the formulation allows the drug to remain in a dissolved state throughout its transit in the GIT. The drug for absorption can be enhanced by formulation of the drug as a solubilizate within a colloidal dispersion. This objective can be achieved by formulation of the drug in a self-emulsifying system. Among various approaches, the self-emulsifying drug delivery system has gained more attention due to enhanced oral bioavailability enabling reduction in dose, more consistent temporal profiles of drug absorption, selective targeting of drug(s) toward specific absorption window in GIT, and protection of drug(s) from the hostile environment in gut [17].

### 2.1. Types of Self-Emulsifying Systems: Self-Emulsifying, Self-Microemulsifying, and Self-Nanoemulsifying Drug Delivery System (SEDDS, SMEDDS, and SNEDDS)

SEDDSs, SMEDDSs and SNEDDSs are physically stable isotropic mixtures of oil, surfactant, cosurfactant, and solubilized drug substance that rapidly and spontaneously form fine oil in water emulsions, microemulsions, or nanoemulsions, respectively, when introduced into aqueous phases under gentle agitation. Thus, self-emulsifying formulations are readily dispersed in the GI tract, where the motility of the stomach and small intestine provides the agitation necessary for emulsification.

The potential advantages of the self-emulsifying systems include 100% drug entrapment capacity, physically stable formulation (can also be filled in capsules), no dissolution step required, formation of submicron droplet size, thus increasing absorption surface area, increase in rate and extent of absorption, and thus increased bioavailability. SEDDS deliver BCS Class II drugs effectively. They also have potential for effective delivery of BCS class III, BCS class IV and hydrolytically susceptible drugs. They provide protection against gastric degradation. Further, they provide consistent temporal profile with reduced dosing, dosing frequency. They are easy to manufacture and scale-up. It also directs the distribution of drug into the lymphatic system.

SEDDSs have been described as systems that produce emulsions with a droplet size between 100 and 300 nm while SMEDDSs form transparent microemulsions with a droplet size of less than 50 nm [20]. However, SEDDS generally refers to all types of self-emulsifying systems unless otherwise described, while SNEDDSs describe systems which form nanoemulsions upon dispersion in aqueous media [21].

When compared with emulsions, which are sensitive and metastable dispersed forms, these self-emulsifying formulations are physically stable, easily manufactured, and are suitable for oral delivery as unit dosage form in soft or hard gelatin capsules due to the anhydrous nature. Thus, for lipophilic drug compounds that exhibit dissolution rate-limited absorption, these systems may offer an improvement in the rate and extent of absorption and result in more reproducible blood-time profiles. Being anhydrous, these systems also offer a great potential for the formulation and administration of hydrolytically susceptible drugs. SEDDS are also found to increase the intestinal permeability and minimize the effect of pH on drug absorption [22].

**Figure 1:**
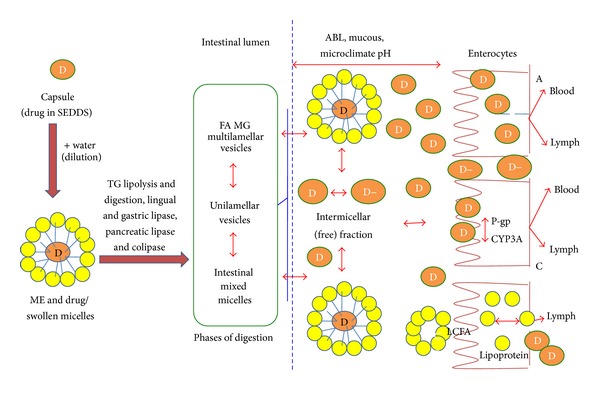
Schematic diagram of intestinal drug transport from lipid-based formulations via the portal and the mesenteric lymphatic routes. (A) Increased membrane fluidity facilitating transcellular absorption. (B) Opening of tight junctions to allow paracellular transport. (C) Inhibition of P-gp and/or CYP450 to increase intracellular concentration and residence time. (D) Stimulation of lipoprotein/chylomicron production. ABL: aqueous boundary layer; D: drug; D−: ionized drug substance; FA: fatty acid; LCFA: long chain fatty acid; ME: microemulsion; MG: monoglyceride; SEDDS: self-emulsifying drug delivery system; TG, triglyceride; TJ, tight junction.

While the primary mechanism by which these formulations are thought to improve drug absorption is through elimination of the need for preabsorptive drug solubilisation in the gastrointestinal tract (GIT), other mechanisms may include protection from chemical and enzymatic degradation localized in the aqueous environment of the GIT and promotion of lymphatic drug transport, which circumvents hepatic first-pass metabolism [2].  Figure 1 gives the schematic diagram of intestinal drug transport from lipid-based formulations via the portal and the mesenteric lymphatic routes [23].

The physicochemical characteristics of the drug substance, the lipid excipients themselves, and the dispersibility of the formulation *in vivo* will determine both the uptake of the drug in the GIT as well as the degree of participation of the portal venous and mesenteric lymphatic pathways in overall drug absorption.

### 2.2. Selection of Excipients in Self-Emulsifying Formulations

The main consideration in selecting appropriate excipients for any lipid-based formulation is in identifying an excipient or their combination having the ability to solubilise the entire drug dose in a volume acceptable for unit oral administration. Self-emulsification has been shown to be specific to the nature of the oil/surfactant pair; the surfactant concentration and oil/surfactant ratio; and the temperature at which self-emulsification occurs [33]. In support of these facts, it has also been demonstrated that only very specific pharmaceutical excipient combinations could lead to efficient self-emulsifying systems. The drug must also be physically and chemically stable in the formulation and the drug release characteristics must remain constant during the shelf life of the formulation. The latter requirement is dependent on the physical and chemical stability of the excipients which must be carefully monitored during formulation development. The excipient should be chosen from the list of generally regarded as safe “GRAS” excipients published by USFDA or from other inactive ingredients approved and published by regulatory agencies. The main excipients in a self-emulsifying system include the lipids (oils), surfactant, and cosurfactant. A few examples of various excipients used in different commercial products are given in Table 3.

#### 2.2.1. Lipids/Oils

The oil represents one of the most important excipients in the self-emulsifying formulations because it can solubilise marked amounts of the lipophilic drug, facilitate self-emulsification, and increase the fraction of lipophilic drug transported via the intestinal lymphatic system, thereby increasing absorption from the GI tract depending on the molecular nature of the triglyceride [16, 46]. Despite the considerable potential that these lipid excipients offer, very few lipid based formulations have reached the pharmaceutical market place. This may be due to the insufficient information regarding the relatively complex physical chemistry of lipids and concerns about formulated drug chemical and physical stability. In addition to these concerns, the interaction of a lipid-based formulation with the GI environment and its impact on drug absorption is also important [2]. For instance, digestible lipids have been shown to be considerably more efficient enhancers of poorly soluble drug absorption, as compared to nondigestible lipids (e.g., liquid paraffin). Fatty acid chain length of the lipid also influences drug absorption [47]. Both long and medium chain triglyceride oils with different degrees of saturation have been used for the design of self-emulsifying formulations. The edible oils which represent the logical and preferred lipid excipient choice for the development of SEDDS are not frequently selected due to their poor ability to dissolve large amounts of lipophilic drugs. Modified or hydrolyzed vegetable oils have been widely used since these excipients form good emulsification systems with a large number of surfactants approved for oral administration and exhibit better drug solubility properties [48]. They offer formulative and physiological advantages, and their degradation products resemble the natural end products of intestinal digestion. Novel semisynthetic medium chain derivatives, which can be defined as amphiphilic compounds with surfactant properties, are progressively and effectively replacing the regular medium chain triglyceride oils in the self-emulsifying drug delivery systems [49].

The lipids exert their effects possibly through several complex mechanisms that can lead to alteration in the biopharmaceutical properties of the drug, such as increased dissolution rate of the drug and solubility in the intestinal fluid, protection of the drug from chemical as well as enzymatic degradation in the oil droplets, and the formation of lipoproteins promoting the lymphatic transport of highly lipophilic drugs [48].

The amount of lipid contained in a formulation also influences the drug absorption primarily via solubilization in the GIT and potentially through activation of GI lipid digestion resulting in increased secretion of pancreatic juice and bile [50]. Thus, the impact of any lipid-based formulation on the lipid digestion processes must be considered, particularly when multiple dosage units of a lipid-based formulation are administered as a single dose, which is common for many anti-HIV drugs.

#### 2.2.2. Surfactants

The self-emulsifying properties require the incorporation of relatively large amounts of surfactant in the formulation in addition to the oily drug carrier vehicle. The surfactants may improve the affinity between lipids and intestinal membrane or increase the permeability of the intestinal membrane. Surfactants increase the permeability by partitioning into the cell membrane and disrupting the structural organization of the lipid bilayer leading to permeation enhancement [51]. Therefore, most drugs are absorbed via the passive transcellular route. They also exert their absorption enhancing effects by increasing the dissolution rate of the drug. Several compounds exhibiting surfactant properties may be employed for the design of self-emulsifying systems, the most widely recommended ones being the nonionic surfactants with a relatively high hydrophilic-lipophilic balance (HLB) values [2, 51, 52]. Safety is a major determining factor in choosing a surfactant. Emulsifiers of natural origin (e.g., lecithin, Akoline medium chain monoglycerides (MCM), and Peceol) are normally preferred since they are considered to be safer than the synthetic surfactants. However, these excipients have limited self-emulsification efficiency [49]. Various vegetable oil derivatives like Acrosyl (castor oil derivative) are still being found to give optimum self-emulsification [53]. Nonionic surfactants are normally preferred over their ionic counterparts due to more favourable safety profiles and greater emulsion stability over a wider range of pH and ionic strength. In addition, nonionic surfactants can produce reversible changes in intestinal mucosal permeability [51], further facilitating absorption of the coadministered drug. Hydrophobic surfactants can penetrate membranes causing changes in membrane fluidity and permeability. Generally single alkyl chains are more penetrative, so bulky surfactants such as polysorbates and triglyceride ethoxylates are found to be less toxic. Usually the surfactant concentration ranges between 30 and 60% of the total formulation in order to form stable SEDDS [33]. It is very important to determine the surfactant concentration properly as large amounts of surfactants may cause GI irritation. However, the extremely small lipid droplet size produced by SMEDDS and SNEDDS formulations promotes rapid stomach emptying and wide dispersion throughout the GIT, minimizing exposure to high local surfactant concentrations and thus reducing the irritation potential. The surfactant involved in the formulation of SEDDS should have a relatively high HLB and hydrophilicity to enable rapid and facile dispersion in the aqueous GI fluid as a very fine oil-in-water emulsion, and hence good self-emulsifying performance can be achieved [2]. The use of surfactant blends to achieve the hydrophilic-lipophilic balance (HLB) value required for emulsification has often been proven to provide superior self-emulsifying properties relative to the use of a single surfactant possessing the desired HLB [54]. One or more cosolvents are often added to the formulation to assist in solubilising high concentrations of the drug. Surfactants are amphiphilic in nature and they can dissolve or solubilize relatively high amounts of hydrophobic drug compounds. There is a relationship between the droplet size and the concentration of the surfactant being used. In many cases, increasing the surfactant concentration could lead to droplets with smaller mean droplet size. This could be explained by the stabilization of the oil droplets as a result of the localization of the surfactant molecules at the oil-water interface. On the other hand, in some cases the mean droplet size may increase with increasing surfactant concentrations [55]. This phenomenon could be attributed to the interfacial disruption elicited by enhanced water penetration into the oil droplets mediated by the increased surfactant concentration and leading to ejection of oil droplets into the aqueous phase. Attempts have been made to evaluate the toxicity of pharmaceutical excipients and SEDDS or SMEDDS formulations *in vitro* in Caco-2 cell monolayers [20, 56].

#### 2.2.3. Cosurfactants

The production of an optimum self-emulsifying formulation requires relatively high concentrations (generally more than 30% w/w) of surfactants. The addition of cosurfactants aids in self-emulsification. The presence of the cosurfactants decreases the bending stress of interface and allows the interfacial film sufficient flexibility to take up different curvatures required to form nanoemulsions over a wide range of composition [57]. Organic solvents such as, ethanol, propylene glycol (PG), and polyethylene glycol (PEG) are suitable for oral delivery, and they enable the dissolution of large quantities of either the hydrophilic surfactant or the drug in the lipid base. On the other hand, alcohols and other volatile cosolvents have the disadvantage of evaporating into the shells of the soft gelatin or hard, sealed gelatin capsules in conventional self-emulsifying formulation leading to drug precipitation. Thus, alcohol-free formulations have also been designed [49], but their lipophilic drug dissolution ability may be limited.

## 3. Mechanism of Self-Emulsification

The mechanism by which self-emulsification occurs is not yet well understood. It has been suggested by Reiss that self-emulsification takes place when the entropy change favouring dispersion is greater than the energy required to increase the surface area of the dispersion [58]. The free energy of a conventional emulsion formulation is a direct function of the energy required to create a new surface between the two phases (oil and water phases) and can be described by

(1) $$\begin{array}{c} \Delta G=\sum_{i}^{}N_{i}\pi r_{i}^{2}\sigma , \end{array}$$

where *G* is free energy associated with the process (ignoring the free energy of mixing),  *N* is the number of droplets, *r* is radius of globules, and *σ* is the interfacial energy.

**Figure 2:**
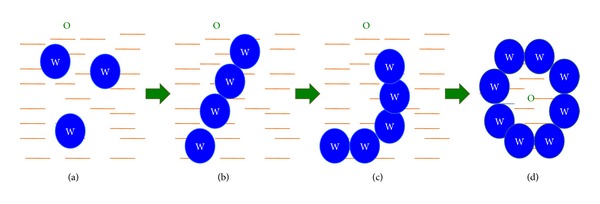
Schematic presentation of the mechanism happening during addition of water in SEDDS. (a) Water droplets in continuous oil phase; (b) water cylinders in oil; (c) lamellar structures; (d) oil droplets in continuous phase.

The two phases of the emulsion tend to separate with time to reduce the interfacial area and thus the free energy of the systems. The conventional emulsifying agents stabilize emulsions resulting from aqueous dilution by forming a monolayer around the emulsion droplets, reducing the interfacial energy and forming a barrier to coalescence. In contrast, emulsification occurs spontaneously with self-emulsifying formulations because the free energy required to form the emulsion is either low and positive or negative [17, 49]. Emulsification requiring very little input energy involves destabilization through contraction of local interfacial regions. It is necessary for the interfacial structure to show no resistance against surface shearing in order for emulsification to take place.  Figure 2 depicts the schematic presentation of the mechanism happening during addition of water in SEDDS in a simplified way.

The ease of emulsification was suggested to be related to the ease of water penetration into the various liquid crystal (LC) or gel phases formed on the surface of the droplet. The interface between the oil and aqueous continuous phases is formed upon addition of a binary mixture (oil/nonionic surfactant) to water. This is followed by the solubilisation of water within the oil phase as a result of aqueous penetration through the interface. This will occur until the solubilisation limit is reached close to the interphase. Further aqueous penetration will lead to the formation of the dispersed LC phase. Eventually, everything that is in close proximity with the interface will be LC, the actual amount of which depends on the surfactant concentration in the binary mixture. Thus, following gentle agitation of the self-emulsifying system, water will rapidly penetrate into the aqueous cores and lead to interface disruption and droplet formation. As a consequence of the LC interface formation surrounding the oil droplets, self-emulsifying formulations become very stable to coalescence. Detailed studies have also been carried out to determine the involvement of the LC phase in the emulsion formation process [31, 33, 59]. Also, particle size analysis and low frequency dielectric spectroscopy (LFDS) were utilized to examine the self-emulsifying properties of a series of Imwitor 742 (a mixture of mono- and diglycerides of capric and caprylic acids)/Tween 80 systems [60]. The results suggested that there might be a complex relationship between LC formation and emulsion formation. Moreover, the presence of the drug compound may alter the emulsion characteristics, probably by interacting with the LC phase. Nevertheless, the correlation between the LC formation and spontaneous emulsification has still not been established.

## 4. Newer Approaches to Self-Emulsifying Drug Delivery System

The self-emulsifying drug delivery systems offers advantages in addressing the challenges of drug solubility and absorption; the next challenge remains the delivery of the drug in an acceptable dosage form. The oral dosage forms are the preferred drug administration route, and lipid formulations offer flexibility for oral dosage forms because they can be formulated as solutions, semisolid, and solid forms. Conventional self-emulsifying drug delivery systems, however, are mostly prepared in a liquid form, which can produce some disadvantages, for example, low stability, irreversible drugs/excipients precipitation, large volume of dose, difficulty in handling and portability, and few choices of dosage forms.

To address these problems, solid-SEDDSs (S-SEDDSs) have been investigated as alternative approaches. Such systems require the solidification of liquid self-emulsifying systems into powders to produce various solid dosage forms (SE capsules, SE tablets, SE pellets, SE beads, and so on). The liquid SEDDS can be converted into solid dosage form without affecting drug release property. Self emulsification happens in GIT by the released contents. Thus, S-SEDDSs combine the advantages of SEDDS (i.e., enhanced solubility and bioavailability) with those of solid dosage forms (e.g., high stability and reproducibility, compact dosage form, ease of handling and portability, and better patient compliance). Knowing the advantages of solid dosage forms, S-SEDDSs have been extensively investigated in recent years, as they frequently correspond to more effective alternatives to conventional liquid SEDDS. Examples include the development of S-SEDDS of Dexibuprofen [36], Nimodipine [45], and Hydrochlorothiazide [28]. From the perspective of dosage forms, S-SEDDSs mean solid dosage forms with self-emulsification properties. S-SEDDSs focus on the incorporation of liquid/semisolid SE ingredients into powders/nanoparticles by different solidification techniques.

The concept of super-SNEDDS of poorly soluble drug Simvastatin has also been investigated. Super-SNEDDSs (200% drug-loaded) were produced by subjecting the SNEDDS preconcentrates to a heating and cooling cycle. The relative bioavailability of the drug from super-SEDDDS was found to increase significantly (180 ± 53.3%) compared to conventional SNEDDS. Prolonged absorption along the small intestine was observed [61]. In one earlier study also, the supersaturatable SEDDS was designed, using a small quantity of HPMC (hydroxy propyl methyl cellulose or other polymers) in the formulation to prevent precipitation of the drug by generating and maintaining a supersaturated state *in vivo*. This system contained a reduced amount of a surfactant, thereby minimizing GI side effects and [62].

For enhancing oral bioavailability of drugs with high solubility and low permeability, water-in-oil-in-water (w/o/w) double emulsions are also investigated. A novel formulation, self-double-emulsifying drug delivery systems (SDEDDSs) were formulated by mixing of hydrophilic surfactants and water-in-oil (w/o) emulsions. SDEDDS can spontaneously emulsify to w/o/w double emulsions in the mixed aqueous gastrointestinal environment, with drugs encapsulated in the internal water phase of the double emulsions [63].

### 4.1. Solidification Techniques for Transforming Liquid SEDDS to Solid-SEDDS (S-SEDDS)

Solid SEDDSs are being developed from liquid/semisolid SEDDS mainly by adsorption on solid carriers [64], spray drying [45], lyophilization [65], melt extrusion [66], and nanoparticle technology. Such powders/nanoparticles, which are referred to as SE nanoparticles/dry emulsions/solid dispersions, are usually further processed into other solid SE dosage forms or, alternatively, filled into capsules (i.e., SE capsules). SE capsules also include those capsules into which liquid/semisolid SEDDSs are directly filled without any solidifying excipient. Other solid SE dosage forms that have emerged in recent years include SE pellets/tablets, SE microspheres/nanoparticles, and SE suppositories/implants [67].

#### 4.1.1. Adsorption on Solid Carriers

Free flowing powders may be obtained from liquid SE formulations by adsorption on solid carriers. The adsorption process is simple and just involves addition of the liquid formulation onto inert carriers and mixing them in a blender. The resulting powder may then be filled directly into capsules or, alternatively, mixed with suitable excipients before compression into tablets. SEDDS can be adsorbed at high levels (up to 70% w/w) onto suitable carriers [64]. Solid carriers can be microporous inorganic substances, high surface-area colloidal inorganic adsorbent substances, cross-linked polymers, or nanoparticle adsorbents, for example, silica, silicates, magnesium trisilicate, magnesium aluminium silicate (Neusilin) microporous calcium silicate (Florite TM RE) magnesium hydroxide, talcum, crospovidone, cross-linked sodium carboxymethyl cellulose, and cross-linked polymethyl methacrylate. The self-emulsifying powder was prepared by adsorbing the liquid SEDDS onto neusilin as carrier to improve the solubility of poorly soluble lercanidipine hydrochloride [35]. Cross-linked polymers create favourable environment to sustain drug dissolution. Nanoparticle adsorbents comprise porous silicon dioxide [36], carbon nanotubes, carbon nanohorns, charcoal, and so forth.

#### 4.1.2. Spray Drying

In this technique, the liquid SEDDS is added to a solution of suitable solid carrier with stirring to obtain the o/w emulsion. This is then atomized into a spray of droplets in a drying chamber, where the volatile phase (e.g., the water contained in an emulsion) evaporates, forming dry particles under controlled temperature and airflow conditions [36, 45]. Such particles can be further prepared into tablets or capsules. The atomizer, the temperature, the most suitable airflow pattern, and the drying chamber design are selected according to the drying characteristics of the product and powder specification. Solid state emulsions are reported by Myers and Shivley (1993). Shivley has used sucrose and mineral oil for preparing solid state emulsions [68].

#### 4.1.3. Lyophilization Technique

Lyophilization or freeze-drying involves transfer of heat and mass to and from the product under preparation. Freeze drying of an oil-in-water emulsion can be an alternative method for the production of dry emulsions. Lyophilization has been thought as a molecular mixing technique where the drug and carrier are codissolved in a common solvent, frozen, and sublimed to obtain a lyophilized molecular dispersion. The potential applications of lyophilization in manufacturing of solid dispersions have successfully been investigated [65, 69, 70]. A slow cooling rate and addition of amorphous cryoprotectants has been reported to have the best stabilizing effects during lyophilization of oil-in-water emulsions [71]. Maltodextrins are also useful matrix forming agent in the formulation of freeze-dried tablets [28].

#### 4.1.4. Melt Granulation

Melt granulation is a technique in which powder agglomeration is obtained through the addition of a lipid as binder that melts or softens at relatively low temperatures. Melt granulation offers several advantages over the conventional wet granulation, since the liquid addition and the subsequent drying phase are omitted. Furthermore, it is also a good alternative to the use of solvent. The main parameters that control the granulation process are impeller speed, mixing time, binder particle size, and the viscosity of the binder. A wide range of solid and semisolid lipids can be applied as meltable binders. For example, Gelucires, a family of vehicles derived from the mixtures of mono-/di-/triglycerides and polyethylene glycols (PEG) esters of fatty acids, is able to increase the dissolution rate compared with PEG usually used before, probably owing to its SE property. Other lipid-based excipients evaluated for melt granulation to create solid SES include lecithin, partial glycerides, or polysorbates. In all cases, the lipidic excipients used must be semisolid at room temperature [66].

#### 4.1.5. Melt Extrusion/Extrusion Spheronization

Melt extrusion is a solvent-free process that allows high drug loading (60%), as well as content uniformity. Extrusion is a procedure of converting a raw material with plastic properties into a product of uniform shape and density, by forcing it through a die under controlled temperature, product flow, and pressure conditions. The size of the extruder aperture will determine the approximate size of the resulting spheroids. The extrusion-spheronization process is commonly used in the pharmaceutical industry to make uniform sized spheroids (pellets). The extrusion-spheronization process requires the following steps: dry mixing of the active ingredients and excipients to achieve a homogenous powder; wet massing with binder; extrusion into rope-like extrudate; spheronization from the extrudate to spheroids of uniform size; drying; sifting to achieve the desired size distribution and coating [34, 66].

### 4.2. Problems Associated with the Solidification Technologies

There are various challenges associated with the solidification technologies. Examples of such problems include the following.

1. Amount of solidifying excipients may affect the release of the drug.
2. Nature of the excipients used may affect the drug absorption.
3. Probability of irreversible phase separation on reconstitution.
4. Clogging of spray nozzles due to oil content in spray-drying method.
5. Degradation of drug during solidification process.
6. Reduction in drug loading capacity.
7. Difficulty in ensuring content uniformity.
8. Probability of residual solvents used during granulation.

### 4.3. Approaches to Overcome the Problems Associated with Solidification Technologies

1. In order to reduce the amount of solidifying excipients required for transformation of SEDDS into solid dosage forms, a gelled SEDDS has been developed. Colloidal silicon dioxide (Aerosil 200) was selected as a gelling agent for the oil-based systems, which served the dual purpose of reducing the amount of required solidifying excipients and aiding in slowing down of the drug release [72].
2. After administration of capsules containing conventional liquid SE formulations, emulsion droplets form and subsequently disperse in the GI tract to reach sites of absorption. However, if irreversible phase separation of the emulsion occurs, an improvement of drug absorption cannot be expected.
  - For handling this problem, sodium dodecyl sulfate was added into the SE formulation [73].
  - With the similar purpose, the supersaturatable SEDDS was designed, using a small quantity of HPMC (or other polymers) in the formulation to prevent precipitation of the drug by generating and maintaining a supersaturated state *in vivo*. This system contains a reduced amount of a surfactant, thereby minimizing GI side effects [62].
3. *Self-emulsifying solid dispersions*. These involve the dispersion of drug in self-emulsifying solid excipients. These excipients have the potential to increase the absorption of poorly water-soluble drugs relative to previously used PEG solid dispersions and may also be filled directly into hard gelatin capsules in the molten state, thus obviating the former requirement for milling and blending before filling. SE excipients like Gelucire1 44/14, Gelucire150/02, Labrasol1, Transcutol1, and tocopheryl polyethylene glycol 1000 succincte (TPGS) have been widely used in this field [10, 11, 26, 69].

## 5. Dosage Forms from Self-Emulsifying Systems

### 5.1. Self-Emulsifying Capsules

Capsule filling is the simplest and the most common technology for the encapsulation of liquid, semisolid, or solid SE formulations for the oral route. The advantages of capsule filling are simplicity of manufacturing, suitability for highly potent low-dose drugs, and high drug loading (up to 50% w/w) potential.

For liquid formulations, it involves a two-step process: filling the formulation into the capsules followed by sealing of the body and cap of the capsule, by banding or microspray sealing. Besides liquid SEDDS filling, the solid-SEDDS obtained by various techniques described above like spray drying, freeze drying, and so forth can be filled in the capsules. After administration of capsules containing conventional liquid SE formulations or the solid-SE formulations, emulsion/nanoemulsion/microemulsion droplets form and subsequently disperse in the GI tract to reach sites of absorption [19].

### 5.2. Self-Emulsifying Tablets

Combinations of lipids and surfactants have presented great potential of preparing SE tablets that have been widely researched. Nazzal et al. developed self-nanoemulsified tablet dosage form of Ubiquinone [67]. First, the self-nanoemulsion system containing the Ubiquinone was prepared; this nanoemulsion was absorbed on granular materials and then compressed to form tablets. Polyethylene oxide successfully illustrated its suitability for controlled-release matrices. The resultant SE tablets consistently maintained a higher active ingredient concentration in blood plasma over the same time frame compared with a nonemulsifying tablet. The newest advance in the research field of SE tablet is the SE osmotic pump tablet, in which the elementary osmotic pump system was chosen as the carrier of SES. This system has outstanding features such as stable plasma concentrations and controllable drug release rate, allowing a bioavailability of 156.78% relative to commercial carvedilol tablets [74].

### 5.3. Self-Emulsifying Sustained/Controlled-Release Pellets

Pellets are multiple unit dosage form which possess many advantages over conventional solid dosage forms, such as flexibility in manufacturing, reduction of intrasubject and intersubject variability of plasma profiles, and minimizing GI irritation without lowering drug bioavailability [75]. Thus, it is very interesting to combine the advantages of pellets with those of SEDDS by SE pellets. SE controlled-release pellets were prepared by incorporating drugs into SES that enhanced their rate of release and then by coating the pellets with a water-insoluble polymer which reduced the rate of drug release. Pellets can be prepared by extrusion/spheronization. The combinations of coating and SES could control *in vitro* drug release and provide a range of release rates [76].

In some investigations, solid self-emulsifying drug delivery systems (solid-SEDDS) were prepared by means of a wet granulation process in a lab-scale high shear mixer in order to improve the dissolution rate of a poorly water-soluble drug. The conventional liquid granulation binder was replaced with an oil-in-water microemulsion, loaded with the drug [34, 42].

### 5.4. Self-Emulsifying Beads

Self-emulsifying system can be formulated as a solid dosage form by using minimum amounts of solidifying excipients. Patil and Paradkar investigated loading SES into the microchannels of porous polystyrene beads (PPB) using the solvent evaporation method. PPB has complex internal void structures typically produced by copolymerizing styrene and divinylbenzene. It is inert and stable over a wide range of pH, temperature and humidity. PPB was found to be potential carriers for solidification of SES, with sufficiently high SES to PPB ratios required to obtain solid form. Bead size and pore architecture of PPB were found to affect the loading efficiency and *in vitro* drug release from SES-loaded PPB [77]. In another study, floating alginate beads containing SEDDS of tetrahydrocurcumin were developed to increase drug solubility and prolong gastric residence time. Use of different proportions of sodium alginate, calcium chloride, and water soluble pore former (polyvinyl alcohol-polyethylene glycol copolymer) in bead formulations was found to have different effects on the floating abilities and *in vitro* drug release rate [78].

### 5.5. Self-Emulsifying Sustained-Release Microspheres

Solid SE sustained-release microspheres were prepared by using the quasi-emulsion-solvent-diffusion method of the spherical crystallization technique. Zedoary turmeric oil (ZTO) exhibited potent pharmacological actions. With ZTO as the oil phase, ZTO release behaviour was controlled by the ratio of hydroxypropyl methylcellulose acetate succinate to Aerosil 200 in the formulation. The plasma concentration-time profiles achieved after oral administration of such microspheres to rabbits showed bioavailability of 135.6% with respect to the conventional liquid SEDDS [79].

### 5.6. Self-Emulsifying Nanoparticles

Nanoparticle techniques are useful in the production of SE nanoparticles. Solvent injection is one of these techniques. In this method, the lipid, surfactant, and drugs are melted together and injected dropwise into a stirred nonsolvent. The resulting SE nanoparticles are filtered out and dried. This approach yielded nanoparticles (about 100 nm) with a high drug loading efficiency of 74% [80].

A second technique is that of sonication emulsion-diffusion-evaporation. The mixture of polylactide-co-glycolide (PLGA) and O-carboxymethyl-chitosan (O-CMC) had a SE effect, with no need to add another surfactant stabilizer. Eventually the 5-FU and plasmid encapsulation efficiencies were found to have 94.5% and 95.7%, respectively, and the 5-FU release activity from the nanoparticles was found to have sustained for three weeks [81].

Trickler et al. developed a novel nanoparticle drug delivery system consisting of chitosan and glyceryl monooleate for the delivery of Paclitaxel. These chitosan/GMO nanoparticles, with bioadhesive properties and increased cellular association, were prepared by multiple emulsion (o/w/o) solvent evaporation methods. The SE property enhanced the solubility of Paclitaxel and provided a foundation for chitosan aggregation, meanwhile causing near 100% loading and entrapment efficiencies of Paclitaxel. These advantages allow the use of lower doses of Paclitaxel to achieve an efficacious therapeutic window, thus minimizing the adverse side effects associated with chemotherapeutics like Paclitaxel [82].

### 5.7. Self-Emulsifying Phospholipid Suspension (SEPS)

Self-emulsifying phospholipid suspension (SEPS) consisting of high amount of phospholipids has the ability to keep the drug in solubilized form *in vivo*, which is essential for bioavailability enhancement. Phospholipids are endogenous lipid with efficient *in vivo* emulsification capability. These require relatively low amount of surfactant/cosurfactant and thus posing less health problems [83].

### 5.8. Self-Emulsifying Suppositories

Some investigators proved that S-SEDDS could increase not only GI adsorption but also rectal/vaginal adsorption [84]. The drugs, which do not easily achieve therapeutic plasma concentrations by oral route, may obtain satisfactory therapeutic levels for chronic hepatic diseases by either vaginal or rectal SE suppositories. There are a few such patented products too.

Self-microemulsifying suppositories of *β*-artemether have been formulated and evaluated with the objective of faster onset of action and prolonged effect when administered by rectal route [85].

### 5.9. Self-Emulsifying Implants

Research into SE implants has greatly enhanced the utility and application of S-SEDDS. As an example, 1,3-bis(2-chloroethyl)-1-nitrosourea (carmustine) is a chemotherapeutic agent used to treat malignant brain tumors. However, its effectiveness was affected by its short half-life. In order to enhance its stability compared with that released from poly(d,l-lactide-co-glycolide) (PLGA) wafer implants, SES was formulated with tributyrin, Cremophor RH 40 (polyoxyl 40 hydrogenated castor oil), and Labrafil 1944 (polyglycolyzed glyceride). Then the self-emulsified carmustine was fabricated into wafers with flat and smooth surface by compression molding. Ultimately, SES increased *in vitro* half-life of carmustine up to 130 min. *In vitro* release of carmustine from SE PLGA wafers was prolonged up to 7 days. Such wafers had higher *in vitro* antitumor activity and were less susceptible to hydrolysis than those wafers devoid of SES [86].

## 6. Physicochemical Characterization Parameters for Self-Emulsifying Formulations

### 6.1. % Transmittance

The primary means of self-emulsification assessment is visual evaluation [87]. To avoid any subjective variations, the % transparency of the resulting micro/nanoemulsion obtained on dilution/reconstitution of the self-emulsifying formulations is measured using UV-visible spectrophotometer [22].

### 6.2. Globule Size and PDI

The globule size of the emulsion is a crucial factor in self-emulsification performance because it determines the rate and extent of drug release as well as absorption [54, 88, 89]. It has been reported that the particle size distribution is one of the most important characteristics of the *in vivo* fate of drug emulsion [37]. The globule size of the reconstituted formulations is most commonly measured using Malvern Zeta Sizer based on the principle of dynamic light scattering (DLS).

### 6.3. Robustness to Dilution

Robustness to dilution is important for SEDDS/SNEDDS to ensure that the emulsion/nanoemulsion formed have similar properties at different dilutions to achieve uniform drug release profile and to ensure that the drug will not get precipitated at higher dilutions *in vivo* which may significantly retard the absorption of the drug from the formulation [49, 90]. The SEDDSs should be evaluated by diluting them at different dilutions and investigating their effect on the properties of the formed emulsion/nanoemulsion [22].

### 6.4. Zeta Potential

This is used to identify the charge of the droplets. The charge of the oil droplets of SEDDS is a property that should be assessed [46]. Generally, the increase in electrostatic repulsive forces between the nanoemulsion droplets prevents the coalescence of nanoemulsion droplets. On the contrary, a decrease of electrostatic repulsive forces causes phase separation. The zeta potential of the reconstituted SEDDS is commonly measured using Malvern Zeta Sizer Nano based on the electrophoresis and electrical conductivity of the formed nanoemulsion.

### 6.5. Effect of pH

The pH of the aqueous phase has considerable influence on the phase behaviour of the spontaneously emulsifying systems [33, 84]. In view of this, the effect of the pH of the aqueous phase on the resultant nanoemulsion should also be investigated.

### 6.6. Effect of Temperature

Self-emulsification has been shown to be specific to the temperature at which self-emulsification occurs [33, 52]. Hence, the effect of temperature on the globule size can also be investigated [22].

### 6.7. Viscosity

The viscosity of the liquid SEDDS is useful to assess its ability to be filled in the hard or soft gelatin capsules. If the system has very low viscosity, it may enhance the probability of leakage from the capsule and the system with too high viscosity may create problem in pourability [91].

### 6.8. Centrifugation Test

This test can be used to determine the stability of the SEDDS after emulsion formation. For this, the samples diluted with distilled water are centrifuged at specified rpm for specified time and then examined for the phase separation [92, 93].

### 6.9. Dye Solubilization Test

The characterization of self-emulsifying drug delivery system can be made utilizing dye solubilization [49]. This test is used to identify the nature of the formed nanoemulsion and its continuous phase. For this, the water-soluble dye is sprinkled onto the surface of the prepared nanoemulsion. By observing the dispersion of dye or the clump formation, the nature of the internal, external phase of the emulsion can be determined.

### 6.10. Cloud Point Measurement

The cloud point is a necessary factor in SEDDS consisting of nonionic surfactants. When the temperature is higher than the cloud point, an irreversible phase separation will occur and the cloudiness of the preparation would have a bad effect on drug absorption, because of the dehydration of its ingredients. Hence, the cloud point for SNEDDS should be above 37°C, which will avoid phase separation occurring in the gastrointestinal tract [22, 94, 95].

### 6.11. Transmission Electron Microscopy

The morphology of the nanoemulsion obtained from SEDDS is investigated using transmission electron microscopy.

## 7. Conclusions

Advancement of the technologies and design and development of new chemical moieties having targeting potential is leading to emergence of new drug molecules having therapeutic effect but unfavourable physicochemical properties for their drug absorption in the body. This is becoming the greatest challenge to the formulation scientists to efficiently deliver such drug molecules mostly exhibiting poor aqueous solubility.

Lipidic formulations are promising approach for various categories of drug molecules having challenging drug properties. Among the various lipid formulations, the self-emulsifying delivery systems offer additional advantages of higher stability, suitability for hydrolytically susceptible drugs, high drug loading capacity, potential for oral drug delivery (solid-SEDDS), ease of manufacture and scale-up, and so forth, if suitably formulated with proper selection of excipients.

SEDDSs are mostly investigated for the BCS class II drugs having low aqueous solubility for their bioavailability enhancement and have shown promising success. They have the potential to solve the problems associated with drugs of all other classes of BCS also as summarized in Table 4. Some studies have already been performed with positive results. More investigations are needed to get better insight in the field.

The challenges associated with the formulation of self-emulsifying system include the selection of right excipients with consideration of their solvent capacity, miscibility, chemical stability, capsule compatibility, self-dispersibility, regulatory issues, and so forth. The major excipients required for their formulation are the oil, surfactant, and the cosurfactant for liquid self-emulsifying systems. The criteria for the selection of the combination of excipients for SEDDS formulations should include their solubilising capacity for the required dose of drug, ability to self-emulsify the system when in contact with the gastric fluid (by use of phase diagram), their regulatory approval state for oral use with consideration of their permitted concentration, and so forth. The material used for transforming liquid SEDDS to solid forms should be inert, compatible and should not affect the emulsifying properties and the release profile of the drug.

The various techniques employed for the solidification may also affect the product quality. Various authors have reported insignificant effect on the emulsification properties of solid-SEDDS prepared by spray drying, free drying (lyophilisation), or the adsorption and extrusion technique with respect to the corresponding liquid SEDDS [34, 36, 45, 65]. Spray drying may be preferred because of their capability to produce smooth surfaced, well separated spherical particles at a rapid rate. But it may not be suitable for thermolabile drugs where lyophilization technique may be beneficial.

Another challenge associated with the SEDDS is that their *in vivo* assessment is difficult in small animals owing to the small volume of gastric fluid in comparison to the humans which may not be sufficient for proper self-emulsification. One can foresee a good scope for the growth of the self-emulsifying drug delivery systems in near future, provided some means are developed for the estimation of their *in vivo* performance. There is a need for the agents which have even better self-emulsifying properties at lower concentrations to minimize any possibility of undesired effects like gastric irritation which may be associated with this system of drug delivery due to comparatively higher amount of surfactant and co-surfactant used in their formulation. A lot of investigations have been done in the field, yet there is a need for the more predictive *in vitro* models for predicting the changes involving the drug in SEDDS in the gut, so that the fate of the drug *in vivo* can be more reliably monitored. Future research may involve human bioavailability studies as well.

## Figures and Tables

**Table 1:** Strategies for the formulation of poorly absorbed drugs.

Technology	Potential advantage	Potential disadvantage	References
Conventional micronization	Known technology, freedom to operate, solid dosage form possible	Poor control of the size distribution of the particles, insufficient improvement in dissolution rate	[24]

Nanocrystals obtained by ball-milling	Established products in the market, experienced technology, solid dosage form possible	Available only under license, secondary process required to avoid aggregation of nanocrystals	[7, 8]

Nanocrystals obtained by dense gas technology	Alternative nanocrystal processing method, still room to develop new IP	Unproven technology, secondary process required to avoid aggregation of nanocrystals	[8]

‘‘Solid solutions”—drug immobilized in polymer	Freedom to operate, new extrusion technology offers solvent-free continuous process, fast and continuous process, low cost	Physical stability of product questionable, possibility of crystallization of drug or polymer	[25]

Self-dispersing ‘‘solid solutions” with surfactants	Steric hindrance to aggregation built into product, amenable to extrusion	Physical stability of product questionable, drug may crystallize	[10, 25, 26]

Nanoparticles and solid lipid nanoparticles	Controlled-release of drug, reduced variability	Low drug loading, drug expulsion after polymorphic transition, high water content	[27]

Lipid solutions (LFCS Type I lipid systems)	GRAS status, simple, safe, and effective for lipophilic actives; drug is presented in solution avoiding the dissolution step, excellent capsule compatibility	Poor solvent capacity, limited to highly lipophilic or very potentdrugs, requires encapsulation	[6, 19]

Self-emulsifying drug delivery systems (SEDDS) and SMEDDS (LCFS Type II or Type III lipid systems)	Prior art available, dispersion leads to rapid absorption and reduced variability, absorption not dependent on digestion	Surfactant may be poorly tolerated in chronic use, soft gel or hard gel capsule can be used but seal must be effective, possible loss of solvent capacity on dispersion (Type III)	[6]

Solid or semisolid SEDDS	Could be prepared as a free flowing powder, filled in capsules or compressed into tablet form, reduced problem of capsule leakage	Surfactant may be poorly tolerated in chronic use, physical stability of product questionable, drug or polymer may crystallize	[25, 28]

Surfactant-cosolvent systems (LFCS Type IV ‘‘lipid” systems)	Relatively high solvent capacity for many drugs (due to surfactant), disperses to micellar solution, reduced variability and irritancy (due to dispersion of surfactant by cosolvent)	Surfactant may be poorly tolerated in chronic use, loss of solvent capacity on dispersion, significant threat of drug precipitation on dilution	[6, 29]

**Table 2:** Lipid formulation classification system.

Parameters	Increasing hydrophilic content →				
	Types of lipid formulations				
	Type I	Type II	Type IIIA	Type IIIB	Type IV
Example/reference	[30]	[31]	[19]	[32]	[29]

Triglycerides or mixed glycerides (%w/w)	100	40–80	40–80	<20	—

Water-insoluble surfactants (%w/w) (HLB < 12)	—	20–60	—	—	0–20

Water-soluble surfactants (%w/w) (HLB > 11)	—	—	20–40	20–50	30–80

Hydrophilic cosolvents (%w/w)	—	—	0–40	20–50	0–50

Particle size of dispersion (nm)	Coarse	250–2000	100–250	50–100	<50

Characteristics	Nondispersing	SEDDS without water-soluble components	SEDDS/SMEDDS with water-soluble components	SMEDDS with water-soluble components and low oil content.	Oil-free formulations

Significance of aqueous dilution	Limited importance	Solvent capacity unaffected	Some loss of solvent capacity	Significant phase changes and potential loss of solvent capacity	—

Significance of digestibility	Crucial requirement	Not crucial but likely to occur	Not crucial but may be inhibited.	Not required and not likely to occur	Not likely to occur

Advantages	GRAS status; simple; excellent capsule compatibility	Unlikely to lose solvent capacity on dispersion	Clear or almost clear dispersion. Absorption without digestion	Clear dispersion. Absorption without digestion	Good solvent capacity for many drugs; disperse to micellar solution

Disadvantages	Poor solvent capacity (unless drug is highly lipophilic)	Turbid o/w dispersion	Possible loss of solvent capacity on dispersion. Less easily digested	Likely loss of solvent capacity on dispersion	Loss of solvent capacity on dispersion; may not be digestible

**Table 3:** Examples of lipids, surfactants, and cosurfactant used in commercial formulations.

Excipient name (commercial name)	Examples of commercial products in which it has been used	References
Lipid ingredients		
Corn oil, mono-, di-, triglycerides, DL-alpha-Tocopherol	Neorol oral solution, Fortavase soft gelatin capsule	[2]
Mixture of mono- and diglycerides of caprylic/capric acid (Akoline)	Avodart soft gelatin capsule	[34]
Triglyceride of the fractionated plant fatty acids C8 und C10 (Miglyol)	Rocaltrol soft gelatin capsule, Hectrol soft gelatin capsule	[34]
Capryol 90 (propylene glycol monocaprylate)		[32, 35, 36]
Captex 355 (glycerol caprylate caprate)		[35]
Capmul MCM L8 (glyceryl mono-dicaprylate1,2,3- propanetriol decanoic acid monoester)		[22, 35]
Oleic acid/ethyl oleate	Ritonavir soft gelatin capsule, Norvir soft gelatin capsule	[37]

Surfactants/cosurfactants		
Polysorbate 20 (Tween 20)	Targretin soft gelatin capsule	[38]
Polysorbate 80 (Tween 80)	Gengraf hard gelatin capsule	[34, 35]
Sorbitan monooleate (Span 80)	Gengraf hard gelatin capsule	[39]
Polyoxy-35-castor oil (Cremophor EL)	Gengraf hard gelatin capsule, Ritonavir soft gelatin capsule	[40]
Polyoxy-40-hydrogenated castor oil (Cremophor RH40)	Nerol soft gelatin capsule, Ritonavir oral solution	[37, 41]
Nikkol HCO-50 (PEG 40 hydrogenated castor oil)		[40]
Polyoxyethylated glycerides (Labrafil M 2125 Cs)	Sandimmune soft gelatin capsules	[36]
Polyoxyethylated oleic glycerides (Labrafil M 1944 Cs)	Sandimmune oral solution	[35, 36, 42, 43]
Labrasol		[36, 37]

Cosurfactants		
Ethanol/glycerine/polypropylene glycol	Nerol, Sandimmune, Lamprene soft gelatin capsule, Nerol oral solution, Gengraf hard gelatin capsule,	
Polyethylene glycol	Targretin soft gelatin capsule, Gengraf hard gelatin capsule, Agenerase soft capsule, Agenerase oral solution	[22, 34, 35]
Transcutol		[40]

Inert adsorbents		
Aerosil 200		[36]
Microcrystalline cellulose		[34]
Neusilin		[44]
Dextran		[43, 45]

**Table 4:** SEDDSs and their potential to solve various problems associated with the drug molecule.

BCS class	Problems proposed to be solved by SEDDS
BCS class I	Enzymatic degradation, acidic degradation, gastric irritation, gut wall efflux
BCS class II	Solubilization, pH dependent solubilization, low bioavailability
BCS class III	Enzymatic degradation, gut wall efflux, low permeability, low bioavailability
BCS class IV	Solubilization, enzymatic degradation, gut wall efflux, low permeability, low bioavailability

## List of Abbreviations

BCS: Biopharmaceutical classification system
FU: Fluorouracil
GIT: Gastrointestinal tract
GRAS: Generally regarded as safe
GMO: Glyceryl monooleate
HLB: Hydrophilic lipophilic balance
LFDS: Low frequency dielectric spectroscopy
PLGA: Polylactic glycolic acid
PPB: Porous polystyrene beads
SE: Self-emulsifying
SEDDS: Self-emulsifying drug delivery systems
SEPS: Self-emulsifying phospholipid suspension
SES: Self-emulsifying systems
SEF: Self-emulsifying formulations
SLN: Solid lipid nanoparticle
SMEDD: Self-microemulsifying drug delivery systems
SNEDD: Self-nanoemulsifying drug delivery systems
ZTO: Zedoary turmeric oil
ABL: Aqueous boundary layer
D: Drug
D−: Ionized drug substance
FA: Fatty acid
LCFA: Long chain fatty acid
ME: Microemulsion
MG: Monoglyceride
TG: Triglyceride
TJ: Tight junction.

## References

[1] Lindenberg M, Kopp S, Dressman JB (2004). Classification of orally administered drugs on the World Health Organization Model list of Essential Medicines according to the biopharmaceutics classification system. *European Journal of Pharmaceutics and Biopharmaceutics*.

[2] Hauss DJ (2007). Oral lipid based Formulations-Enhancing the Bioavailablity of Poorly water soluble drugs. *Drugs and Pharmaceutical Sciences*.

[3] Kawakami K (2012). Modification of physicochemical characteristics of active pharmaceutical ingredients and application of supersaturatable dosage forms for improving bioavailability of poorly absorbed drugs. *Advanced Drug Delivery Reviews*.

[4] Lipinski CA (2000). Drug-like properties and the causes of poor solubility and poor permeability. *Journal of Pharmacological and Toxicological Methods*.

[5] Lipinski CA, Lombardo F, Dominy BW, Feeney PJ (2001). Experimental and computational approaches to estimate solubility and permeability in drug discovery and development settings. *Advanced Drug Delivery Reviews*.

[6] Pouton CW (2006). Formulation of poorly water-soluble drugs for oral administration: physicochemical and physiological issues and the lipid formulation classification system. *European Journal of Pharmaceutical Sciences*.

[7] Merisko-Liversidge E, Sarpotdar P, Bruno J (1996). Formulation and antitumor activity evaluation of nanocrystalline suspensions of poorly soluble anticancer drugs. *Pharmaceutical Research*.

[8] Merisko-Liversidge E, Liversidge GG, Cooper ER (2003). Nanosizing: a formulation approach for poorly-water-soluble compounds. *European Journal of Pharmaceutical Sciences*.

[9] Kaushal AM, Gupta P, Bansal AK (2004). Amorphous drug delivery systems: molecular aspects, design, and performance. *Critical Reviews in Therapeutic Drug Carrier Systems*.

[10] Serajuddln ATM (1999). Solid dispersion of poorly water-soluble drugs: early promises, subsequent problems, and recent breakthroughs. *Journal of Pharmaceutical Sciences*.

[11] Sethia S, Squillante E (2003). Solid dispersions: revival with greater possibilities and applications in oral drug delivery. *Critical Reviews in Therapeutic Drug Carrier Systems*.

[12] Shulman M, Cohen M, Soto-Gutierrez A (2011). Enhancement of naringenin bioavailability by complexation with hydroxypropoyl-*β*-cyclodextrin. *PLoS ONE*.

[13] Anwar M, Warsi MH, Mallick N (2011). Enhanced bioavailability of nano-sized chitosan-atorvastatin conjugate after oral administration to rats. *European Journal of Pharmaceutical Sciences*.

[14] Divyakant BP, Valay RM, Alok NT, Arpita AP, Hetal PT (2012). Development and characterization of solid lipid nanoparticles for enhancement of oral bioavailability of Raloxifene. *Journal of Pharmacy and Bioallied Sciences*.

[15] Aungst BJ (2000). Intestinal permeation enhancers. *Journal of Pharmaceutical Sciences*.

[16] Nielsen FS, Petersen KB, Müllertz A (2008). Bioavailability of probucol from lipid and surfactant based formulations in minipigs: influence of droplet size and dietary state. *European Journal of Pharmaceutics and Biopharmaceutics*.

[17] Gursoy RN, Benita S (2004). Self-emulsifying drug delivery systems (SEDDS) for improved oral delivery of lipophilic drugs. *Biomedicine and Pharmacotherapy*.

[18] Schwendener RA, Schott H (1996). Lipophilic 1-*β*-D-arabinofuranosyl cytosine derivatives in liposomal formulations for oral and parenteral antileukemic therapy in the murine L1210 leukemia model. *Journal of Cancer Research and Clinical Oncology*.

[19] Pouton CW (2000). Lipid formulations for oral administration of drugs: non-emulsifying, self-emulsifying and ’self-microemulsifying’ drug delivery systems. *European Journal of Pharmaceutical Sciences*.

[20] Gursoy N, Garrigue J-S, Razafindratsita A, Lambert G, Benita S (2003). Excipient effects on *in vitro* cytotoxicity of a novel paclitaxel self-emulsifying drug delivery system. *Journal of Pharmaceutical Sciences*.

[21] Mahmoud EA, Bendas ER, Mohamed MI (2009). Preparation and evaluation of self-nanoemulsifying tablets of carvedilol. *AAPS PharmSciTech*.

[22] Gupta S, Chavhan S, Sawant KK (2011). Self-nanoemulsifying drug delivery system for adefovir dipivoxil: design, characterization, in vitro and ex vivo evaluation. *Colloids and Surfaces A*.

[23] O’Driscoll CM (2002). Lipid-based formulations for intestinal lymphatic delivery. *European Journal of Pharmaceutical Sciences*.

[24] Joshi JT (2011). A review on micronization techniques. *Journal of Pharmaceutical Sciences and Research*.

[25] Kolter K, Karl M, Gryczke A (2012). *Hot Melt Extrusion with BASF Pharma Polymers—Extrusion Compendium*.

[26] Serajuddin ATM, Sheen P-C, Mufson D, Bernstein DF, Augustine MA (1988). Effect of vehicle amphiphilicity on the dissolution and bioavailability of a poorly water-soluble drug from solid dispersions. *Journal of Pharmaceutical Sciences*.

[27] Musicanti C, Gasco P (2012). Solid lipid nanoparticle. *Encyclopedia of Nanotechnology*.

[28] Corveleyn S, Remon JP (1997). Formulation and production of rapidly disintegrating tablets by lyophilisation using hydrochlorothiazide as a model drug. *International Journal of Pharmaceutics*.

[29] Strickley RG (2004). Solubilizing excipients in oral and injectable formulations. *Pharmaceutical Research*.

[30] Tokumura T, Tsushima Y, Tatsuishi K (1987). Enhancement of the oral bioavailability of cinnarizine in oleic acid in beagle dogs. *Journal of Pharmaceutical Sciences*.

[31] Pouton CW (1997). Formulation of self-emulsifying drug delivery systems. *Advanced Drug Delivery Reviews*.

[32] Seo YG, Kim DH, Ramasamy T (2013). Development of docetaxel-loaded solid self-nanoemulsifying drug delivery system (SNEDDS) for enhanced chemotherapeutic effect. *International Journal of Pharmaceutics*.

[33] Pouton CW (1985). Self-emulsifying drug delivery systems: assessment of the efficiency of emulsification. *International Journal of Pharmaceutics*.

[34] Iosio T, Voinovich D, Perissutti B (2011). Oral bioavailability of silymarin phytocomplex formulated as self-emulsifying pellets. *Phytomedicine*.

[35] Kallakunta VR, Bandari S, Jukanti R, Veerareddy PR (2012). Oral self emulsifying powder of lercanidipine hydrochloride: formulation and evaluation. *Powder Technology*.

[36] Balakrishnan P, Lee B-J, Oh DH (2009). Enhanced oral bioavailability of dexibuprofen by a novel solid Self-emulsifying drug delivery system (SEDDS). *European Journal of Pharmaceutics and Biopharmaceutics*.

[37] Yi T, Wan J, Xu H, Yang X (2008). A new solid self-microemulsifying formulation prepared by spray-drying to improve the oral bioavailability of poorly water soluble drugs. *European Journal of Pharmaceutics and Biopharmaceutics*.

[38] Song WH, Park JH, Yeom DW (2013). Enhanced dissolution of celecoxib by supersaturating self-emulsifying drug delivery system (S-SEDDS) formulation. *Archives of Pharmacal Research*.

[39] Mercuri A, Belton PS, Royall PG (2012). Identification and molecular interpretation of the effects of drug incorporation on the self-emulsification process using spectroscopic, micropolarimetric and microscopic measurements. *Molecular Pharmacology*.

[40] Singh B, Singh R, Bandyopadhyay S (2013). Optimized nanoemulsifying systems with enhanced bioavailability of carvedilol. *Colloids and Surfaces B*.

[41] Niederquell A, Kuentz M (2013). Proposal of stability categories for nano-dispersions obtained from pharmaceutical self-emulsifying formulations. *International Journal of Pharmaceutics*.

[42] Franceschinis E, Bortoletto C, Perissutti B, Dal Zotto M, Voinovich D, Realdon N (2011). Self-emulsifying pellets in a lab-scale high shear mixer: formulation and production design. *Powder Technology*.

[43] Oh DH, Kang JH, Kim DW (2011). Comparison of solid self-microemulsifying drug delivery system (solid SMEDDS) prepared with hydrophilic and hydrophobic solid carrier. *International Journal of Pharmaceutics*.

[44] Hentzschel CM, Alnaief M, Smirnova I, Sakmann A, Leopold CS (2012). Enhancement of griseofulvin release from liquisolid compacts. *European Journal of Pharmaceutics and Biopharmaceutics*.

[45] Nicolaos G, Crauste-Manciet S, Farinotti R, Brossard D (2003). Improvement of cefodixim poxetil oral absorption in rats by an oil-in-water submicron emulsion. *Int J Pharm*.

[46] Gershanik T, Benita S (2000). Self-dispersing lipid formulations for improving oral absorption of lipophilic drugs. *European Journal of Pharmaceutics and Biopharmaceutics*.

[47] Deckelbaum RJ, Hamilton JA, Moser A (1990). Medium-chain versus long-chain triacylglycerol emulsion hydrolysis by lipoprotein lipase and hepatic lipase: implications for the mechanisms of lipase action. *Biochemistry*.

[48] Hauss DJ, Fogal SE, Ficorilli JV (1998). Lipid-based delivery systems for improving the bioavailability and lymphatic transport of a poorly water-soluble LTB4 inhibitor. *Journal of Pharmaceutical Sciences*.

[49] Constantinides PP (1995). Lipid microemulsions for improving drug dissolution and oral absorption: physical and biopharmaceutical aspects. *Pharmaceutical Research*.

[50] Khoo S-M, Shackleford DM, Porter CJH, Edwards GA, Charman WN (2003). Intestinal lymphatic transport of halofantrine occurs after oral administration of a unit-dose lipid-based formulation to fasted dogs. *Pharmaceutical Research*.

[51] Swenson ES, Milisen WB, Curatolo W (1994). Intestinal permeability enhancement: efficacy, acute local toxicity, and reversibility. *Pharmaceutical Research*.

[52] Wakerly MG, Pouton CW, Meakin BJ (1986). Self emulsification of vegetable oil-non-ionic surfactant mixtures. *ACS Symposium Series*.

[53] Shah SR, Parikh RH, Chavda JR (2013). Self-nanoemulsifying drug delivery system of glimepiride: design, development, and optimization. *PDA Journal of Pharmaceutical Science and Technology*.

[54] Wang L, Dong J, Chen J, Eastoe J, Li X (2009). Design and optimization of a new self-nanoemulsifying drug delivery system. *Journal of Colloid and Interface Science*.

[55] Kommuru TR, Gurley B, Khan MA, Reddy IK (2001). Self-emulsifying drug delivery systems (SEDDS) of coenzyme Q10: formulation development and bioavailability assessment. *International Journal of Pharmaceutics*.

[56] Gershanik T, Haltner E, Lehr C-M, Benita S (2000). Charge-dependent interaction of self-emulsifying oil formulations with Caco-2 cells monolayers: binding, effects on barrier function and cytotoxicity. *International Journal of Pharmaceutics*.

[57] Solans C, Izquierdo P, Nolla J, Azemar N, Garcia-Celma MJ (2005). Nano-emulsions. *Current Opinion in Colloid and Interface Science*.

[58] Reiss H (1975). Entropy-induced dispersion of bulk liquids. *Journal of Colloid And Interface Science*.

[59] Pouton CW, Wakerly M, Meakin BJ (1987). Self-emulsifying systems for oral delivery of drugs. * International Symposium on Control Release Bioactive Materials*.

[60] Craig DQM, Lievens HSR, Pitt KG, Storey DE (1993). An investigation into the physico-chemical properties of self-emulsifying systems using low frequency dielectric spectroscopy, surface tension measurements and particle size analysis. *International Journal of Pharmaceutics*.

[61] Thomas N, Holm R, Garmer M (2013). Supersaturated self-nanoemulsifying drug delivery systems (Super-SNEDDS) enhance the bioavailability of the poorly water-soluble drug simvastatin in dogs. *AAPS Journal*.

[62] Gao P, Rush BD, Pfund WP (2003). Development of a supersaturable SEDDS (S-SEDDS) formulation of paclitaxel with improved oral bioavailability. *Journal of Pharmaceutical Sciences*.

[63] Qi X, Wang L, Zhu J, Hu Z, Zhang J (2011). Self-double-emulsifying drug delivery system (SDEDDS): a new way for oral delivery of drugs with high solubility and low permeability. *International Journal of Pharmaceutics*.

[64] Ito Y, Kusawake T, Ishida M, Tawa R, Shibata N, Takada K (2005). Oral solid gentamicin preparation using emulsifier and adsorbent. *Journal of Controlled Release*.

[65] El-Badry M, Fathy M (2006). Enhancement of the dissolution and permeation rates of meloxicam by formation of its freeze-dried solid dispersions in polyvinylpyrrolidone K-30. *Drug Development and Industrial Pharmacy*.

[66] Verreck G, Brewster ME (2004). Melt extrusion-based dosage forms: excipients and processing conditions for pharmaceutical formulations. *Gattefossé Bulletin Technique*.

[67] Nazzal S, Smalyukh II, Lavrentovich OD, Khan MA (2002). Preparation and *in vitro* characterization of a eutectic based semisolid self-nanoemulsified drug delivery system (SNEDDS) of ubiquinone: mechanism and progress of emulsion formation. *International Journal of Pharmaceutics*.

[68] Myers SL, Shively ML (1992). Preparation and characterization of emulsifiable glasses: oil-in-water and water-in-oil-in-water emulsions. *Journal of Colloid and Interface Science*.

[69] Betageri GV, Makarla KR (1995). Enhancement of dissolution of glyburide by solid dispersion and lyophilization techniques. *International Journal of Pharmaceutics*.

[70] Fathy M, Sheha M (2000). In vitro and in vivo evaluation of an amylobarbitone/hydroxypropyl-*β*- cyclo-dextrin complex prepared by a freeze-drying method. *Pharmazie*.

[71] Bamba J, Cave G, Bensouda Y, Tchoreloff P, Puisieux F, Couarraze G (1995). Cryoprotection of emulsions in freeze-drying: freezing process analysis. *Drug Development and Industrial Pharmacy*.

[72] Patil P, Joshi P, Paradkar A (2004). Effect of formulation variables on preparation and evaluation of gelled self-emulsifying drug delivery system (SEDDS) of ketoprofen. *AAPS PharmSciTech*.

[73] Itoh K, Tozuka Y, Oguchi T, Yamamoto K (2002). Improvement of physicochemical properties of N-4472 part I formulation design by using self-microemulsifying system. *International Journal of Pharmaceutics*.

[74] Wei L, Li J, Guo L (2007). Investigations of a novel self-emulsifying osmotic pump tablet containing carvedilol. *Drug Development and Industrial Pharmacy*.

[75] Abdalla A, Mäder K (2007). Preparation and characterization of a self-emulsifying pellet formulation. *European Journal of Pharmaceutics and Biopharmaceutics*.

[76] Serratoni M, Newton M, Booth S, Clarke A (2007). Controlled drug release from pellets containing water-insoluble drugs dissolved in a self-emulsifying system. *European Journal of Pharmaceutics and Biopharmaceutics*.

[77] Patil P, Paradkar A (2006). Porous polystyrene beads as carriers for self-emulsifying system containing loratadine. *AAPS PharmSciTech*.

[78] Sriraksa S, Sermkaew N, Setthacheewakul S (2012). Floating alginate beads as carriers for self-emulsifying system containing tetrahydrocurcumin. *Advanced Materials Research*.

[79] You J, Cui F-D, Han X (2006). Study of the preparation of sustained-release microspheres containing zedoary turmeric oil by the emulsion-solvent-diffusion method and evaluation of the self-emulsification and bioavailability of the oil. *Colloids and Surfaces B*.

[80] Attama AA, Nkemnele MO (2005). In vitro evaluation of drug release from self micro-emulsifying drug delivery systems using a biodegradable homolipid from Capra hircus. *International Journal of Pharmaceutics*.

[81] Yunxia H, Jin C, Yi G, Xubo Y, Chunsheng K, Peiyu P (2005). Preparation and evaluation of 5-FU/PLGA/gene nanoparticles. *Key Engineering Materials*.

[82] Trickler WJ, Nagvekar AA, Dash AK (2008). A novel nanoparticle formulation for sustained paclitaxel delivery. *AAPS PharmSciTech*.

[83] Shanmugam S, Park J-H, Kim KS (2011). Enhanced bioavailability and retinal accumulation of lutein from self-emulsifying phospholipid suspension (SEPS). *International Journal of Pharmaceutics*.

[84] Kim JY, Ku YS (2000). Enhanced absorption of indomethacin after oral or rectal administration of a self-emulsifying system containing indomethacin to rats. *International Journal of Pharmaceutics*.

[85] Gugulothu D, Pathak S, Suryavanshi S, Sharma S, Patravale V (2010). Self-microemulsifiyng suppository formulation of *β*-artemether. *AAPS PharmSciTech*.

[86] Gang SC, Jin SL, Seon HK (2005). Enhancement of the stability of BCNU using self-emulsifying drug delivery systems (SEDDS) and in vitro antitumor activity of self-emulsified BCNU-loaded PLGA wafer. *International Journal of Pharmaceutics*.

[87] Craig DQM, Barker SA, Banning D, Booth SW (1995). An investigation into the mechanisms of self-emulsification using particle size analysis and low frequency dielectric spectroscopy. *International Journal of Pharmaceutics*.

[88] Shah NH, Carvajal MT, Patel CI, Infeld MH, Malick AW (1994). Self-emulsifying drug delivery systems (SEDDS) with polyglycolyzed glycerides for improving in vitro dissolution and oral absorption of lipophilic drugs. *International Journal of Pharmaceutics*.

[89] Tarr BD, Yalkowsky SH (1989). Enhanced intestinal absorption of cyclosporin in rats through the reduction of emulsion droplet size. *Pharmaceutical Research*.

[90] Date AA, Nagarsenker MS (2007). Design and evaluation of self-nanoemulsifying drug delivery systems (SNEDDS) for cefpodoxime proxetil. *International Journal of Pharmaceutics*.

[91] Patil P, Patil V, Paradkar A (2007). Formulation of a self-emulsifying system for oral delivery of simvastatin: in vitro and in vivo evaluation. *Acta Pharmaceutica*.

[92] Patel PA, Chaulang GM, Akolkotkar A (2008). Self emulsifying drug delivery system. *Research Journal of Pharmacy and Technology*.

[93] Shafiq S, Shakeel F, Talegaonkar S, Ahmad FJ, Khar RK, Ali M (2007). Development and bioavailability assessment of ramipril nanoemulsion formulation. *European Journal of Pharmaceutics and Biopharmaceutics*.

[94] Zhang P, Liu Y, Feng N, Xu J (2008). Preparation and evaluation of self-microemulsifying drug delivery system of oridonin. *International Journal of Pharmaceutics*.

[95] Elnaggar YSR, El-Massik MA, Abdallah OY (2009). Self-nanoemulsifying drug delivery systems of tamoxifen citrate: design and optimization. *International Journal of Pharmaceutics*.

